# Natural genetic variation for fruit set rate within Malbec grapevine (*Vitis vinifera* L.) clones

**DOI:** 10.1186/s12870-025-06660-1

**Published:** 2025-05-08

**Authors:** Luciano Calderón, Silvina van Houten, Claudio Muñoz, Tomás Oroño, Laura Bree, Daniel Bergamin, Cristóbal Sola, Diana Segura, Natalia Carrillo, Sebastián Gómez-Talquenca, José Miguel Martinez-Zapater, Javier Tello, Diego Lijavetzky

**Affiliations:** 1 Instituto de Biología Agrícola de Mendoza (CONICET-UNCuyo), Mendoza, Argentina; 2 Facultad de Ciencias Agrarias, Universidad Nacional de Cuyo, Mendoza, Argentina; 3 Vivero Mercier Argentina, Mendoza, Argentina; 4 Instituto Nacional de Tecnología Agropecuaria (INTA) EEA Mendoza, Mendoza, Argentina; 5 Instituto de Ciencias de la Vid y del Vino (ICVV; CSIC, Gobierno de La Rioja, Universidad de La Rioja), Logroño, Spain

**Keywords:** Malbec, Clonal diversity, Reproductive performance, Yield component, Somatic mutation, Flower development

## Abstract

**Background:**

Fruit set is the transformation of flower ovaries into berries. Fruit set rate determines the number of berries produced per bunch, which is a major component of yield in grapevines. Malbec is a black-berried grapevine cultivar recognized for producing high-quality wines, and particularly relevant for Argentina’s winemaking industry. Clonal variation has been reported for Malbec at the phenotypic and molecular levels. However, less is known about clonal variation for agronomically relevant features affecting yield. In this work we evaluated 25 Malbec clones for the fruit set rate and other related features over multiple seasons.

**Results:**

The mean fruit set rate was 38.6% (ranged between 13.1% and 65.8%) in 2021/22, and 32.1% (ranged between 9.8% and 50%) in 2022/23 season, evidencing a wide range of within-season variation. Besides the expectable interannual differences, significant and positive correlations were found over seasons for the fruit set rate, number of flowers per inflorescence and number of berries per bunch. Moreover, multivariate clustering analyses consistently grouped the evaluated clones into three distinct groups. Two of these groups showed similar number of flowers per inflorescence, but significantly different fruit set rates and number of berries per bunch. Representative clones of the latter two groups were in-depth analyzed in 2023/24, supporting previous seasons results and revealing differences in their floral phenotypes.

**Conclusions:**

We found a wide range of clone-dependent variation for the evaluated traits, which generated great differences in the reproductive performance and yield within Malbec. We hypothesized that the observed differences were associated to somatic mutations, affecting the correct development and functionality of flower organs in a clone-specific way.

**Supplementary Information:**

The online version contains supplementary material available at 10.1186/s12870-025-06660-1.

## Background

Grapevine production extends over 7.3 mha of vineyards worldwide, primarily sustaining the winemaking industry which produced *ca*. 258 mhl of wine in 2023 [1]. Argentina is one of the main wine exporters of the world [1] and more than half of these exported wines include Malbec, either as single varietal or in blends [2]. Malbec is a black-berried grapevine cultivar of French origin, that was introduced to the Mendoza region (Argentina) in the 1850 s [3]. This cultivar has shown great phenotypic plasticity since its introduction to Mendoza region, producing high-quality wines under different growing conditions [4]. Accordingly, several studies have reported clonal diversity within Malbec at the phenotypic level, including fruit composition [5] and phenology [6]. Also, epigenetic [7], genotypic [8], and transcriptomic diversity [9] have been reported within Malbec. However, the natural clonal variation that exists for agronomically relevant traits associated with yield remains poorly understood.

A stable production of good quality fruit is always sought by grape producers. Thus, it is key to understand how different factors affect grapevine yield and quality. The number of berries per bunch is one of the major components explaining seasonal variation in yield. This trait is the outcome of a series of complex mechanisms (i.e. pollination, fertilization, and fruit set) that ultimately determine the overall reproductive performance of a plant [10]. Fruit set is the transformation of the quiescent ovary of the flower into the dynamically growing berry [11]. Consequently, the fruit set rate measures the proportion of flowers that have turned into berries in the same inflorescence/bunch, after the successful completion of the pollination and fertilization events [11]. In grapevine, the fruit set rate is fully determined one or two weeks after flowering time, as the delayed drop of fruitlets is rare [12]. Nonetheless, there are different reproductive disorders (e.g. coulure and millerandage) that might occur during and after flowering time, that can reduce the final number of fully developed seeded berries in a bunch. Coulure (also known as shatter) occurs when an excessive number of flowers or recently set berries shed, producing low number of seeded berries of normal size in the mature bunch. Millerandage describes the situation when an excessive number of small seedless berries and/or live green ovaries (LGOs) are observed in the mature bunch, compared to the number of normal seeded berries [13]. Thus, specific indexes have been proposed for a more precise and quantitative description of the mentioned disorders [14].

Multiple factors affect the fruit set rate and fruit set-related traits in grapevine, including the prevalent weather conditions during key phenological stages. For example, high temperature events during bud formation induces a lower number of flowers per inflorescence [15], whereas cold weather conditions at flowering time reduce fertilization and pollination rates [16]. Fruit set is also affected by environmental factors associated with soil nutrition [17] and soil salinity [18]. To overcome such difficulties, different cultural practices have been assayed to increase grape production, including nitrogen supply [19] or growth regulators usage [14]. Also, shoot tipping [14], late pruning [20], as well as trunk and shoot girdling [21]. Even though the described practices have been proved effective at improving grapevines fruit set rate and yield, they are time-consuming and cost-expensive.

Grapevine cultivars vary in their fruit set rate and reproductive performance. This cultivar-dependent variation hold across different growing regions and seasons [10, 22, 23], making evident the genetic control regulating such traits [24]. Grapevine cultivars are highly heterozygous organisms that must be clonally propagated to preserve their varietal attributes. During this process, spontaneous somatic mutations might generate phenotypic changes, that might be relevant for cultivar improvement [25], including yield-related traits [16, 26, 27]. Therefore, it is important to screen the available natural variation that exists in plant material collections. Such screenings would allow to select the proper plant material (i.e. clone, cultivar) to mitigate yield-limiting climate conditions, in a more sustainable way [28, 29]. Also, identifying and comparing individuals with extreme phenotypes can be useful to unravel the molecular causes underlying the observed variation in agronomically relevant features [30].

In this work, we have analyzed the natural intra-varietal variation for the fruit set rate and other fruit set-related traits within Malbec. The screening of a large collection of clones revealed a wide range of intra-varietal diversity. The implemented approach allowed us to identify several somatic variants of interest, and to hypothesized that clone-specific mutations affecting flowers development and functionality could be responsible for the observed differences in reproductive performance and yield.

## Materials and methods

### Plant material

We analyzed 25 Malbec clones, each represented by three different plants (biological replicates). All plants are grown in the same experimental vineyard at Vivero Mercier Argentina (Perdriel, Mendoza, Argentina; −33.09° S, −68.87° W), where they were planted in 2001. The experimental vineyard is under the same environmental conditions and maintained under even cultural practices. The vineyard is located in a silty loam soil, planted following series of North–South orientated rows (2 m between rows, and 1 m between vines). Vines are trained in a bilateral cordon and protected with anti-hail nets (white polyethylene). Vineyard is drip-irrigated to soil capacity, 5 mm/day between Oct-Nov and Feb-Apr and 7 mm/day during Dic-Jan (when highest temperatures occur in the experimental site). The varietal identity of all the evaluated clones was previously determined, and they were assigned to a clonal genotype [8], as indicated in Supp. Table 1.

The sanitary status of all the analyzed plants was evaluated, because certain virus combinations are known to reduce reproductive performance and yield [31]. We tested for the presence of the nine most frequently reported grapevine viruses in the region of Mendoza (GLRaV-1 to 4, GVA, GVB, GFKV, RASPaV, and GFLV) [32]. For each clone, total RNA was isolated from each of the three biological replicates and pooled into a composite sample for qRT-PCR screening. Only in case of a positive result the three individual samples were separately analyzed.

### Phenotypic evaluations

#### Fruit set rate and fruit set-related variables analyses

Phenotypic evaluation of the 25 clones of Malbec was conducted in two consecutive seasons (2021/22 and 2022/23). Five basal inflorescences of five independent shoots were selected per plant (so, 15 inflorescences per clone), tagged and bagged with a nylon mesh bag following Ibáñez et al. [23] one week before flowering (E-L stages 17–18, according to the modified grapevine growth scale) [33]. Mesh bags were removed after fruit set completion, when berries achieved pea-size (E-L stage 31). Subsequently, the bags content (mainly flower caps and dropped unfertilized ovaries) was carefully spread onto the glass surface of a HP-Deskjet-F4180 scanner and digitalized to obtain one image per inflorescence. Only occasionally, more images were obtained to avoid content overlapping. These images were used to obtain the number of flowers per inflorescence, considering that each flower has one single flower cap (i.e., calyptra) [13]. To this aim, RGB scanned images (751 in total) were automatically analyzed with the software FIJI [34], employing a customized version of the macro developed by [23]. Modifications introduced to customize the image processing protocol for flower caps counting are detailed in the Additional file 1, and available at https://github.com/jvtello/Fruitset_Malbec. Correlation analyses between the automatically and manually counted images were performed to validate the automated process (Fig. S1).

Total soluble solids were measured after *veraison* every three days on berries of the non-tagged bunches of the evaluated plants, with a PAL-1 ATAGO refractometer (Atago, Tokyo, Japan), in wt/wt of sucrose and expressed in Brix degrees (°Bx). The tagged bunches were harvested when berries reached an average of 23°Bx (E-L stage 38) and transported to the laboratory for further analysis. Bunch weight was determined with a scale (Ranger 300, Ohaus, Pine Brook, NJ, USA) and bunches were manually destemmed to obtain the total number of berries per bunch (BN), considering colored berries. Fruit set rate (FS) was calculated as indicated by [14]:

$$\text{Fruitset} \left(\%\right)=\frac{\text{Berries per bunch}}{\text{Flowers per bunch}}\times 100$$

#### Detailed phenotypic evaluation of clones with contrasting fruit set rates

Four clones showing contrasting features in 2021/22 and 2022/23 seasons were selected for an extended phenotypic characterization in a third season (2023/24). Aiming to reduce the effect that the number of flowers per inflorescence has on the fruit set rate [14], we focused on clones with a non-significantly different number of flowers (see Results). We selected clones MB-505 and MB-508 for showing high fruit set rate and high number of berries per bunch, whereas clones MB-510 and MB-515 were selected for showing low fruit set rate and low number of berries per bunch. We analyzed three plants per clone and six inflorescences per plant, which were tagged and bagged at E-L stages 17–18 as described previously. In 2023/24 three additional inflorescences per plant were sampled at E-L stage 23 (full-bloom) for the visual evaluation of flowers structural and functional anomalies, and pollen viability (in one inflorescence). For pollen viability assessment, four recently opened flowers from the mid-section of the inflorescence were selected [35]. Pollen grains were stained using the Alexander’s modified method [36], which provides a good correlation with pollen germination rates [37]. Preparations were observed using a Nikon Eclipse E200 microscope (Nikon, Tokyo, Japan), and RGB images from the stained pollen grains were obtained using a Zeiss AxioCam 208 camera (Carl Zeiss, Göttingen, Germany). Image contrast and saturation was adapted to optimize the visual differentiation between viable and non-viable pollen grains. In some cases, several images were needed from one preparation to reach an average of 1,000 pollen grains per inflorescence, obtaining a total of 561 RGB images. Viable and non-viable pollen grains were counted with the software FIJI [34] using the semi-automated system developed by Tello et al. [35], customized for this work and available at https://github.com/jvtello/Fruitset_Malbec. Modifications introduced to the image processing protocol for pollen viability analysis are fully described in the Additional file 1, alongside with the correlation analysis done between automated and manual data for its validation (Fig. S1). Pollen viability was also assessed for the other 21 clones evaluated in previous seasons, for a wider overview.

The evaluation of flower structural/functional anomalies for the four selected clones was based on ca. 100 flowers per inflorescence, and three inflorescences per plant (adding nine inflorescences per clone). Flowers were separated from the inflorescence and individually inspected using a Nikon SMZ800 magnifying glass (Nikon, Tokyo, Japan). Images of individual flowers were taken with a Zeiss AxioCam 208 camera. The percentage of normal and anomalous flowers was obtained by direct observation, as well as the number of flowers showing indehiscent and/or half opened anthers with evident unreleased pollen grains.

After fruit set completion (stage E-L 31), mesh bags were removed from the inflorescences to analyze their content. Different to previous seasons, in 2023/24 we separated and counted manually the unfertilized dropped ovaries before scanning the flower caps. The number of flower caps was assessed automatically, through the image-based system detailed above. Mature bunches were collected at harvest time (E-L stage 38) to evaluate the same yield components than in previous seasons and bunch compactness, which was assessed following the descriptor 204 from the International Organization of Vine and Wine (OIV) [38]. Bunches were manually destemmed and berries were differentially counted as seeded berries, seedless berries, and live green ovaries (LGOs) [20]. These counts allowed the estimation of the fruit set rate (as previously described), as well as coulure (CI) and millerandage (MI) indexes [14] as follows:

$$\text{Coulure Index }\left(\text{CI}\right)=10-\frac{\left(\text{Seeded berries}+\text{Seedless berries}+\text{LGOs}\right)\times 10}{\text{Flowers}}$$

$$\text{Millerandage Index }\left(\text{CI}\right)=10-\frac{\left(\text{Seeded berries}\right)\times 10}{\text{Seeded berries}+\text{Seedless berries}+\text{LGOs}}$$

The number of seeds per bunch and the mean number of seeds per berry were calculated by dissecting all seeded berries. Finally, all seeds were weighted to calculate seed mean weight, and the empty seeds rate was estimated by floatability in water, as an indicator of non-viable seed rate production [39].

### Statistical analyses

All statistical analyses were conducted in R v. 4.4 (http://www.r-project.org) using the required tools and packages. Pearson’s correlation coefficients (r) between all the evaluated traits were calculated as described in [40], considering significant results at *p* ≤ 0.05. Principal component analyses (PCA) and hierarchical clustering on principal components (HCPC) were performed using the *FactoMineR* v. 2.8 [41] package. Clones clustering results were visualized using the *factoextra* v. 1.0 package [42]. Analyses of variance (ANOVA) were performed using the *stats* package embedded in R to assess for phenotypic differences, either between groups of clones (according to HCPC results) or individual clones. Tukey HSD post-hoc tests were conducted to test for significant differences, and results were considered statistically significant at *p* ≤ 0.05.

## Results

### Fruit set rate, reproductive performance and yield-related traits exhibit a wide range of clonal variation in Malbec

All the composite samples resulted positive for RSPaV, considered to be a highly prevalent but benign virus in the Mendoza region [32]. Only four clones (MB-46, MB-509, MB-598, and MB-712) showed positive results for other viruses (Supp. Table 2). The individual analyses of the three plants of these four samples revealed positive results for two different plants of MB-46 (Supp. Table 2). Regarding MB-509, MB-598 and MB-712, only one plant turned positive just for the GFkV virus (Supp. Table 2). Since none of the 25 clones turned positive for a combination of viruses potentially compromising its reproductive performance, all were considered for phenotyping assessments (Supp. Table 2).

**Fig. 1:**
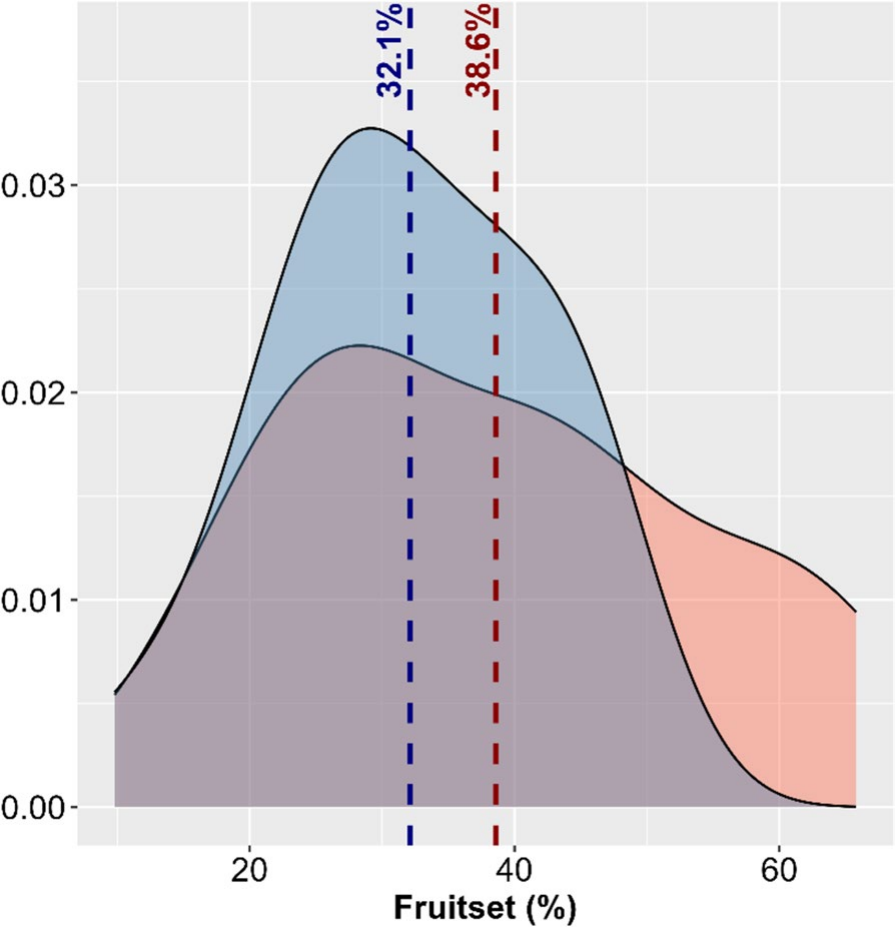
Density plot of the fruit set rate variation observed within Malbec, based on 25 clones analyzed in 2021/22 (red) and 2022/23 (blue). Seasonal average values are shown as dashed lines

The phenotypic evaluation of 25 Malbec clones revealed a wide range of variation for all the analyzed traits, both between and within seasons. As expected, differences in the fruit set rates were observed when the between-seasons mean values were compared, being 38.4 ± 15% in 2021/22 and 32.1 ± 10% in 2022/23 (Fig. 1). Within-seasons, the fruit set rate exhibited high clonal variation, ranging from 13.1% (MB-715) to 65.8% (MB-508) in 2021/22, and from 9.8% (MB-510) to 50.1% (MB-508) in 2022/23 (Supp. Table 1). The mean number of flowers per inflorescence in 2022/23 was 260.2 ± 82, slightly higher than that observed in 2022/21 (201.2 ± 77). The number of flowers per inflorescence also exhibited high level of within-seasons clonal variation, ranging between 112.4 ± 4.4 (MB-509) and 360.5 ± 15.8 (MB-46) in 2021/22, and between 153.9 ± 8.6 (MB-713) and 501.9 ± 18.4 (MB-42) in 2022/23 (Supp. Table 1). The mean number of berries per bunch was 77.2 ± 29 in 2022/23, higher than in 2021/22 (70.1 ± 30.1). The number of berries per bunch also showed a wide range of within-seasons clonal variation, spanning from 30.9 ± 10.5 (MB-715) to 141 ± 47.1 (MB-46) in 2021/22, and from 23.1 ± 13.2 (MB-510) to 140.4 ± 30.5 (MB-46) in 2022/23 (Supp. Table 1). Despite interannual differences, positive and significant correlations were observed between traits (Fig. S2).

**Fig. 2:**
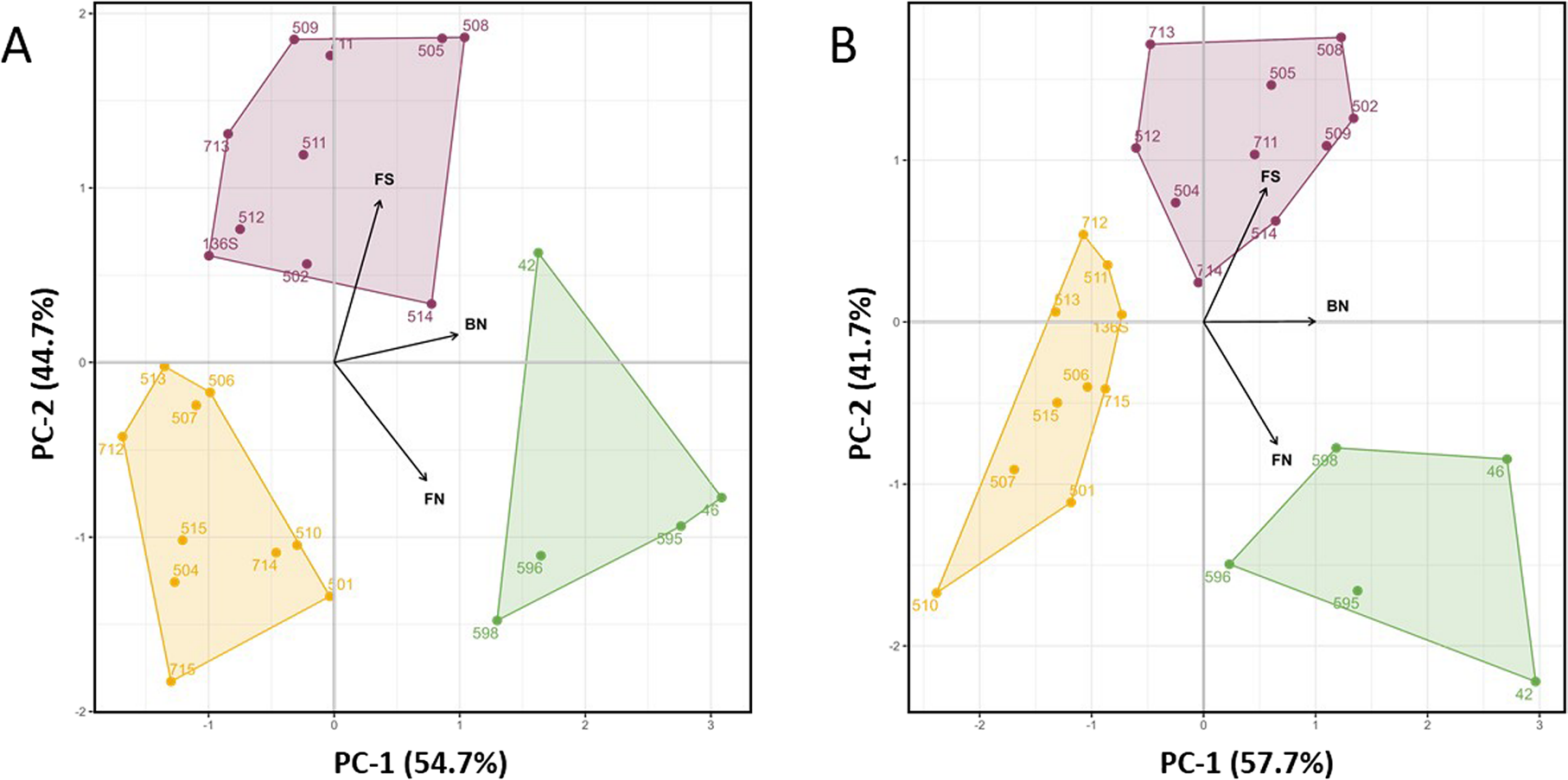
The hierarchical clustering on principal components analysis shows that the 25 Malbec clones clustered in the same three groups in two consecutive seasons, **A** 2021/22 and **B** 2022/23. Multivariate analyses were based on the variables, FS = Fruit set (%), BN = Number of berries per bunch and FN = Number of flowers per inflorescence. The color-code employed to assign clones to each group is Yellow, Green and Purple and this code is held along the manuscript

Multivariate analyses were performed for each season, including all screened clones and based on three variables of interest: fruit set rate, flowers per inflorescence, and berries per bunch. Bunch weight was excluded from multivariate analyses due to its total correlation with the number of berries per bunch (*r* = 1) (Fig. S2). PCA tests retrieved similar results in both seasons (Fig. 2). PC-1 explained the greatest percentage of the variance, and it was positively associated with the number of berries per bunch (BN). PC-2 revealed the opposite relation between the fruit set (FS) and the number of flowers per inflorescence (FN). Additionally, the HCPC analyses showed that the 25 clones clustered consistently in three groups. As color-coded in Fig. 2, the yellow and purple groups included ten clones each, while the green group included five clones. In agreement with the high correlation observed for all the evaluated traits (Fig. S2), most clones revealed a consistent cluster assignment between seasons (Fig. 2). Only four clones (MB-714, MB-504, MB-511 and MB-136S) switched clusters between seasons (Fig. 2 and Supp. Table 1).

**Fig. 3:**
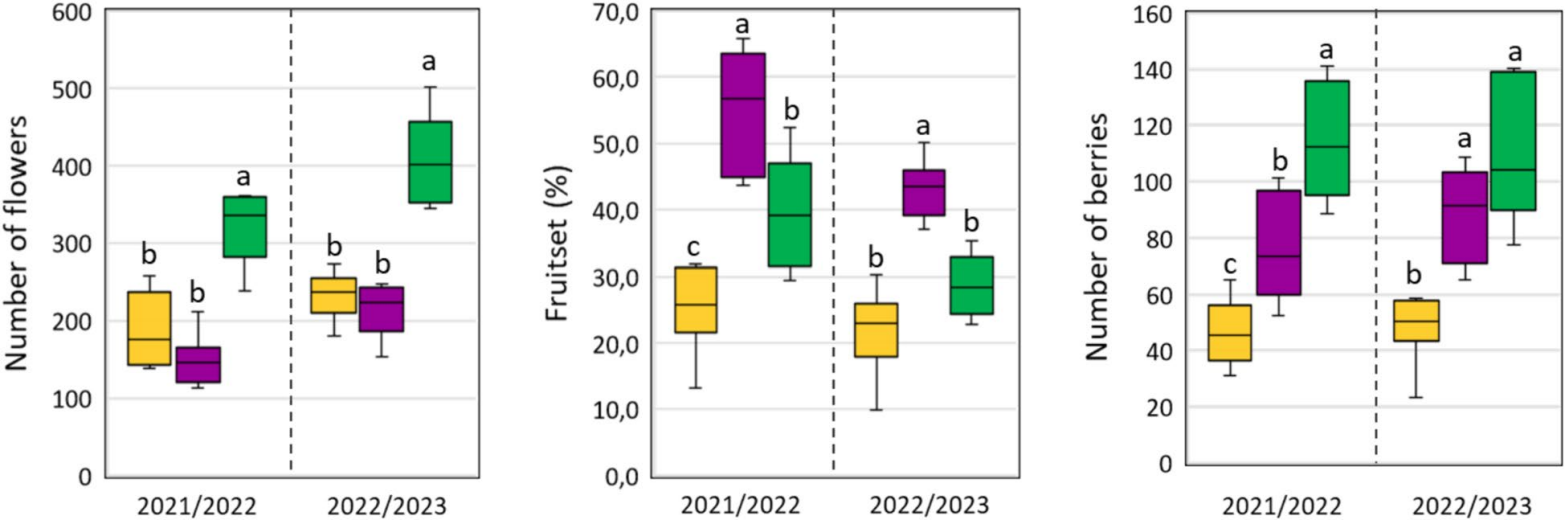
Boxplots showing the phenotypic distribution for the number of flowers, fruit set rate (%), and the number of berries of the 25 clones. Clones were compared based on the three groups resulting from the HCPC and the boxplots color-code is also based on the HCPC results. Different lowercase letters indicate significant differences among groups within-season, Tukey’s HSD test (*p* ≤ 0.05)

ANOVAs also revealed consistent results among the three groups of clones for the mean values of the evaluated features, although some differences between groups were not statistically significant (Fig. 3). The green group of clones had the highest mean number of flowers per inflorescence in both seasons (323.9 ± 49 and 403.6 ± 61 in 2021/22 and 2022/23, respectively), followed by the yellow (189.2 ± 48 and 233.3 ± 29) and purple (148.8 ± 32.6 and 215.4 ± 33.6) groups (Fig. 3). The three groups behaved consistently also for the fruit set rate in both seasons, where the purple group showed the highest mean values (54.7 ± 9% in 2021/22 and 43.2 ± 4% in 2022/23), followed by the green (39.2 ± 8% and 28.5 ± 4%) and the yellow (25.2 ± 6% and 21.56 ± 6%) groups (Fig. 3). Regarding the number of berries per bunch, the green group of clones exhibited the highest mean values in both seasons (114.9 ± 21 in 2021/22 and 112.5 ± 26 in 2022/23), followed by the purple (77.1 ± 18 and 89.2 ± 16) and yellow (46.4 ± 11 and 48.5 ± 11) groups (Fig. 3). Consistent with this observation, the green group of clones exhibited the highest mean value for the whole bunch weight, followed by the purple and yellow groups (Fig. S3).

### Malbec clones with reduced reproductive performance exhibited alterations in their flower development and/or functionality

**Fig. 4:**
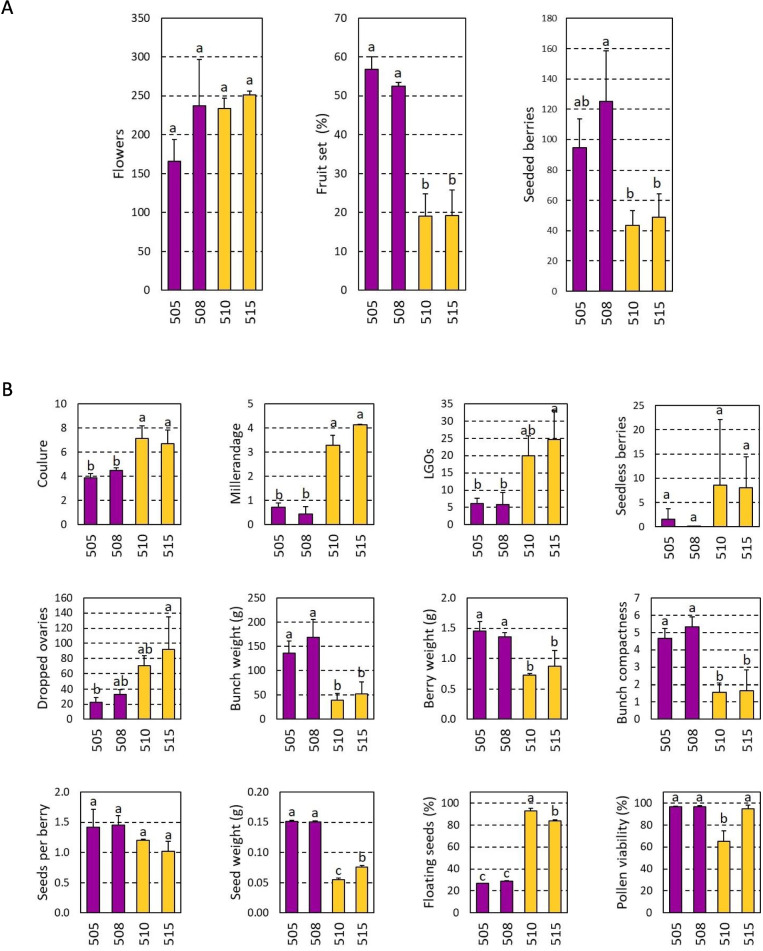
In-depth analysis of the four selected clones in 2023/24 season. **A** Shows the three traits that were analyzed in two previous seasons for all clones. **B** Shows traits analyzed only for these four clones. Bar-charts are color-coded according to Fig. 2, with the two clones representing the high fruit set and yield group in purple (MB-505 and MB-508), and the two clones representing the low fruit set and yield group in yellow (MB-510 and MB-515). Lowercase letters indicate significant differences, Tukey HSD test (*p* ≤ 0.05)

Results obtained in 2023/24 for MB-505, MB-508, MB-510 and MB-515 were consistent with the observations obtained in previous seasons. We found that these four clones do not differ significantly in the number of flowers per inflorescence, but MB-505 and MB-508 had higher fruit set rates and number of berries per bunch than MB-510 and MB-515 (Fig. 4A). Both MB-510 and MB-515 showed significantly higher coulure and millerandage indexes values than MB-505 and MB-508, due to their higher number of seedless berries and LGOs (Fig. 4B). The number of dropped ovaries (i.e. unfertilized flowers) was also higher in MB-510 and MB-515 than in MB-505 and MB-508 (Fig. 4B). In consequence, MB-505 and MB-508 showed significantly higher bunch weight and bunch compactness values than MB-510 and MB-515 (Fig. 4B). Lastly, the number of seeds per berry did not differ significantly between these four clones, but seeds of MB-510 and MB-515 were significantly lighter and exhibited a greater floatability index than those of MB-505 and MB-508 (Fig. 4B).

**Fig. 5:**
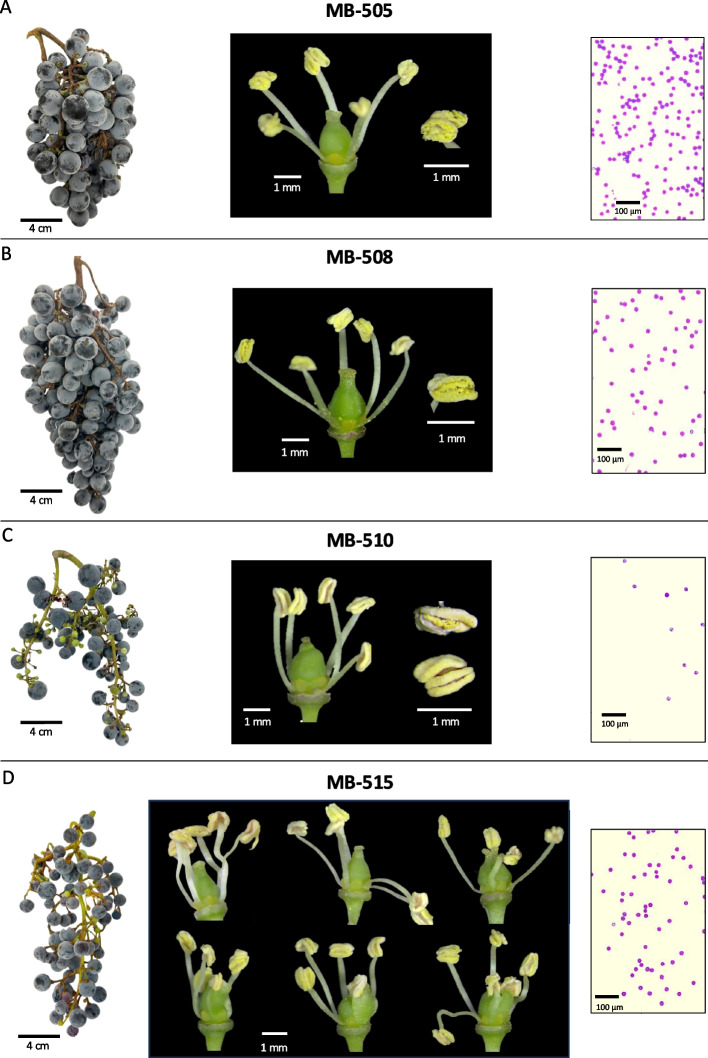
Images of a typical bunch (left), flower features (center) and a representative capture of the Alexander-stained preparations for pollen viability assessment (right), are presented for the four deeply analyzed clones. In pollen preparations dark-stained grains are viable and light-stained are non-viable. **A** MB-505 and **B** MB-508 had typical bunch features for Malbec and predominantly well-formed hermaphrodite flowers in their inflorescences. Pollen grains were abundant and mostly viable. **C** MB-510 and **D** MB-515 showed loose bunches for different reasons. In **C** flowers were hermaphrodite and well-formed but anthers remained indehiscent or half opened at full-bloom, releasing few and mostly non-viable pollen grains. In **D** a high incidence of flower malformations was observed, although anthers functionality and pollen features were similar to (**A**) and (**B**)

Pollen viability assessments did not reveal significant differences between the two high-yielding clones (MB-505 and MB-508) and the low-yielding clone MB-515, but there were significant differences with MB-510 (Fig. 4B). In fact, MB-510 was the only one with a markedly low pollen viability (65.0 ± 9.5%) when we evaluated this trait in the 25 Malbec clones (Fig. S4). At the same time, we observed some potential limitations of pollen release in MB-510, as observed from the few pollen grains observed in the Alexander-stained preparations (Fig. 5C).

The visual inspection of MB-505, MB-508, MB-510 and MB-515 flowers revealed some structural and functional anomalies, particularly in clones with reduced reproductive performance. In this regard, MB-505 and MB-508 showed the typical structures expected in the hermaphrodite flowers of cultivated grapevines, with five stamens showing fully erect filaments with dehiscent anthers, and one pistil of regular shape and size (Fig. 5A and B). On the other hand, we found that more than 54% of MB-510 flowers showed half-opened and/or completely indehiscent anthers (Fig. 5C). This was evidenced in the low number of pollen grains observed during pollen viability analyses (Fig. 5C). Anther’s indehiscence remained the same throughout the entire flowering period for MB-510, even until flower senescence (data not shown). Besides, MB-515 exhibited a high incidence of flowers with aberrant phenotypes. Visual observations revealed that 52% of its flowers had one or different combinations of abnormal floral structures. Within them, we observed flowers with crinkled and/or semi-reflexed stamens, flowers with fused stamens and carpe, flowers with aberrant carpel and nectary development, and flowers with an unusual number of stamens (four and six) (Fig. 5D). However, MB-515 flowers did not exhibit apparent problems with its male reproductive system, since neither pollen release nor viability seemed affected when compared to other clones (Figs. 4B and 5).

## Discussion

### Malbec clones exhibit a wide range of natural genetic variation for fruit set rate

The accurate assessment of fruit set rate in grapevines requires precise evaluation of both the number of flowers and the number of berries in the same inflorescence/bunch [22]. Whereas the manual counting of berries is straightforward, the manual counting of flowers is a time-consuming task when many samples are to be analyzed. Here, we used a customized semi-automated approach, for studying a large collection of clones. By employing this approach, we explored Malbec fruit set rate considering for both clonal and interannual variation. Evaluations were conducted in Mendoza (Argentina), the region with the largest vineyard surface of Malbec in the world [43]. Previous works catalogued Malbec as a medium-to-poor fruit set cultivar (with values around 30%), based on few observations obtained in vineyards of California (USA) [44] and in Logroño (Spain) [23]. Similar reduced fruit set rates were reported in a commercial vineyard of Malbec from Valle de Uco (Mendoza, Argentina) [21]. In agreement to previous reports, the mean fruit set rate estimated here for 25 clones ranged between 30 and 40%, which is slightly below the fruit set value considered normal for wine grape cultivars (50%) [12, 23]. Therefore, our results confirm Malbec as a cultivar with reduced fruit set, similar to other wine cultivars of international relevance like Cabernet Sauvignon and Merlot [22].

A significant contribution of the present work is the finding of a wide range of phenotypic variation within Malbec clones for traits of agronomic interest that have a direct impact on seasonal yield. We found clones with very low fruit set rates (less than 10%), whereas others had values over 60%. Similar ranges of variation for fruit set have been observed when different grapevine cultivars have been compared [22–24]. Here, we also report a large variation for other related traits, like the number of flowers per inflorescence and the number of berries per bunch. At the same time, clones exhibited a consistent performance between seasons, confirming the strong genetic control determining fruit set-related traits [16, 24]. Wide ranges of clonal variation for fruit set have been previously reported for other cultivars, like Garnacha Tinta [45] and Tempranilllo Tinto [16, 27]. All these findings indicate that fruit set rate depends not only on the cultivar, but also on the clone. Such variation is a valuable asset for grape growers to adapt their viticulture practices to market needs, and to face climate change-related challenges [28, 29].

Multivariate analyses divided the 25 clones in three groups of similar phenotypic characteristics, with little impact of seasonal variation (Fig. 2). Within them, the green group was consistently conformed by the same five clones in the two evaluated seasons. These clones had the highest number of berries per bunch. Based on a previous genotyping screening [8], these five clones were found to belong to the same clonal lineage, with a short history of vegetative propagation in Argentina (Supp. Table 1) that can be traced back to 1990s (C. Sola, personal comm.). Our findings support the common origin of these clones, which have undergone similar selection criteria. These five clones were originally prospected by ENTAV-INRA® during the 1970s across old vineyards in south-western France (https://www.plantgrape.fr/), when the aim of clonal selection programs was to obtain virus-free and high-yielding plants [46]. Therefore, the high reproductive performance observed in these five clones might be reflecting those early clonal selection targets.

On the other hand, the yellow and purple groups clustered Malbec clones that did not differ significantly in their number of flowers per inflorescence. These clones were prospected during the 1990s in vineyards of the Mendoza region. These prospections followed a selection program focused on finding low-yielding clones of optimum fruit composition (C. Sola, personal comm.) to produce high quality wines [47]. Agreeing with these selection targets, clones of the purple and yellow groups were found to have lower number of flowers and berries per bunch than clones of the green group. Interestingly, the purple and yellow groups had an overrepresentation of genotypes of the Malbec clonal lineage with a long history of vegetative propagation in Argentina [8] (Supp. Table 1). This finding indicates that the vegetative propagation undergone by Malbec in Argentina has generated somatic variations with impact on its reproductive performance and yield. Altogether, the combination of genetic and phenotypic data revealed the contrasting targets aimed in clonal selection programs (high yield vs. fruit quality) sought in France and Argentina at different times.

### Somatic variations affecting flowers functionality and development might explain low fruit set rates within Malbec clones

Genetic variations affecting fruit set rate can be caused by different factors, including gamete viability and germination [18, 24]. Pollen and ovules need both to be viable to trigger seeds and berries development [39]. Here, pollen viability assessments indicated that most Malbec clones had values above 75%, enough to ensure adequate fertilization processes [35]. Only MB-510 presented a significantly lower value (65.0 ± 9.5%), potentially being the main factor hindering its reproductive performance [16]. Nonetheless, the pollen viability value observed for MB-510 might not be low enough to explain its reduced fruit set rate, since only one viable pollen grain is needed to fertilize each ovule [48]. Interestingly, MB-510 was also found to be deficient at pollen release, likely derived from the high percentage of its flowers showing indehiscent or half-opened anthers. Besides, pollen features of MB-515 were rather normal and not different to those of the high-fruit set clones used as control (MB-505 and MB-508). However, the underlying cause of its reduced capacity to set fruits might be on the high rate of aberrant flowers detected. Malbec flowers show the typical floral structures of most cultivated grapevines [49]. In MB-515, we found multiple anomalous flower structures, including reflexed, semi-reflexed and/or crinkled stamens’ filaments. These types are common in flowers of female *V. vinifera* ssp. *sylvestris* individuals [49], which usually produce non-viable [49] and/or fewer [50] pollen grains than flowers of hermaphrodite grapevine cultivars. Nonetheless, pollen formation and development does not seem to be affected in MB-515, and we did not detect differences on the main features (viability and abundance) of the pollen independently obtained from MB-515 normal and aberrant flowers (data not shown).

Overall, MB-510 and MB-515 exhibit some genetically-determined limitations to set fruit that ultimately reduce the number of fully developed berries and seeds at harvest time, and reduce bunch compactness and yield. In addition, MB-510 and MB-515 berries had a high percentage of floater seeds, another evidence of reproductive dysfunctions [51]. However, the genetic and molecular mechanisms with this convergent phenotypic effect are likely to be different in both clones. In this regard, it would be of interest to analyze if the molecular mechanisms previously described in grapevine somatic variants with altered male fertility are also present in MB-510 [52, 53]. Likewise, MB-515 flowers shows some of the flower alterations previously described in a somatic variant of grapevine cv. Bouchalès with abnormal petal and stamen structures [54, 55]. This phenotype was associated to a skewed expression pattern of the *VvPI* (also known as *VvMADS9*) gene, a transcription factor that regulates the early-stages of grapevine flower differentiation and development [55]. Therefore, the evaluation of the expression levels of *VvPI* and other floral homeotic genes might be useful to reveal their involvement on the unusual floral structures observed in MB-515.

## Supplementary Information

Additional file 1. Supplementary methods. Detailed description of the modifications introduced to the semi-automated protocols employed for flower caps counting and pollen viability determination.

Additional file 2: Supplementary Table 1. Phenotypic variation for 25 Malbec clones, during two seasons (2021/22 and 2022/23). The mean and standard deviation (±) observed for four evaluated traits are shown (FN: flowers number; BN: berries number; FS: fruit set %, BuW: whole bunch weight). Rows color code follows the HCPC results for clones clustering. Grey color indicates clones that switched clusters between seasons. Genotype and lineage information is provided for each clone.

Additional file 3: Supplementary Table 2. A) Sanitary status of the 25 Malbec clones analyzed, based on composite samples (three plants) screened for the nine most frequent viruses found in Mendoza region (Argentina). The (-) symbol indicates a negative result and (+) symbol indicate positive results. Highlighted in red are positive samples for infrequent viruses in Mendoza region. B) Detailed results of the individual samples that showed positive results in the first screening.

Additional file 4: Figure S1. Correlations between manual and automated counting for custom modifications introduced to the semi-automated protocols. Number of flower caps per inflorescence (A), total number of pollen grains per image (B), and viable pollen grains per image (C).

Additional file 5: Figure S2. Pearson’s correlation coefficients (r) obtained for the five evaluated traits for 25 clones in two consecutive seasons. Squares size and color vary according to correlation coefficients (see blue-to-red scale in the bottom). White squares indicate non-significant correlation values between variables (*p*-value < 0.05).

Additional file 6: Figure S3. Boxplots showing the phenotypic distribution for bunch weight, based on the clonal groups indicated by HCPC analyses. Groups of clones are colored as indicated in Fig. 2. Different lowercase letters indicate significant differences among groups per season (2021/22 and 2022/23), according to a Tukey HSD test (*p* ≤ 0.05).

Additional file 7: Figure S4. Bar plots showing the pollen viability (%) values obtained for the 25 Malbec clones, during 2023/24. Each bar represents a clone colored according to HPCP results (see Fig. 2). Clones in grey showed inconsistent clustering results between seasons. The dashed line indicates the 80% threshold, above which grapevines are considered to have -high to very high- pollen viability. Malbec-510 was the only clone below that threshold.

## Data Availability

All data generated or analyzed during this study are included in this published article [and its supplementary information files].
