# Supplementary material for: Natural genetic variation for fruit set rate within Malbec grapevine (Vitis vinifera L.) clones
Source: BMC Plant Biol. 2025 May 8;25:606. doi: 10.1186/s12870-025-06660-1 (PMC12060385; doi:10.1186/s12870-025-06660-1)
Supplement: Supplementary file 1 — Additional file 1. Supplementary methods. Detailed description of the modifications introduced to the semi-automated protocols employed for flower caps counting and pollen viability determination. [file 12870_2025_6660_MOESM1_ESM.docx]

**Additional File 1.** Supplementary methods. Detailed description of the modifications introduced to the semi-automated protocols employed for flower caps counting and pollen viability determination.

**Number of flowers per inflorescence counting:** The customized semi-automated tool splits each RGB image into its three primary channels (red, green and blue). Then, the red channel-derived image is binarized into a black and white image using Yen’s automatic multilevel thresholding approach (Yen et al., 1995). The resulting regions of interest (ROIs, flower caps in this case) were automatically counted using the command “Analyze Particles” of FIJI, considering only the ROIs with a surface area ranging between 200 and 1,000 pixels^2^. This automatic approach was validated using a random subset of 80 images, for which the number of flower caps was also manually counted. The positive and significant correlation obtained between the manually and automatically counts supported the use of the automatized approach for the aim of this work (Fig. S1). Then, a linear regression model between the automatic and manual values was calculated to obtain the estimated number of flowers (FN) for all the analyzed inflorescences.

**Pollen viability determination**: The customized version of this semi-automated method incorporates a color deconvolution process (Ruifrok & Johnston, 2001), as implemented in the Color Deconvolution 2 plugin for FIJI (Landini et al., 2021). We used the Giemsa method built-in stain vector, as its staining components (methylene blue and eosin) resemble the optical properties of the main components of Alexander’s stain (malachite green and acid fuchsin) (Peterson et al., 2010). After this step, the deconvolved images were used to address the automated counting of total and viable pollen grains. In both cases, pollen grains were counted only if they had a surface area from 60 to 800 pixels2, and a circularity (calculated from diameter data) from 0.40 to 1.00. In parallel, the number of total and viable pollen grains was manually counted in a random subset of 84 images. The high and significant correlation obtained between both approaches (automated and manual) for both counts (total and viable pollen grains) supported the usage of the automated process (Fig. S1)

**References**

Landini, G., Martinelli, G., & Piccinini, F. (2021). Colour deconvolution: Stain unmixing in histological imaging. *Bioinformatics*, *37*(10), 1485-1487. https://doi.org/10.1093/bioinformatics/btaa847

Peterson, R., Slovin, J. P., & Chen, C. (2010). A simplified method for differential staining of aborted and non-aborted pollen grains. *International Journal of Plant Biology*, *1*(2), Article 2. https://doi.org/10.4081/pb.2010.e13

Ruifrok, A. C., & Johnston, D. A. (2001). Quantification of histochemical staining by color deconvolution. *Analytical and quantitative cytology and histology*, *23*(4), 291-299.

Yen, J.-C., Chang, F.-J., & Chang, S. (1995). A new criterion for automatic multilevel thresholding. *IEEE Transactions on Image Processing*, *4*(3), 370-378. IEEE Transactions on Image Processing. https://doi.org/10.1109/83.366472
