# Supplementary material for: Natural genetic variation for fruit set rate within Malbec grapevine (Vitis vinifera L.) clones
Source: BMC Plant Biol. 2025 May 8;25:606. doi: 10.1186/s12870-025-06660-1 (PMC12060385; doi:10.1186/s12870-025-06660-1)
Supplement: Supplementary file 3 — Additional file 3: Supplementary Table 2. A) Sanitary status of the 25 Malbec clones analyzed, based on composite samples (three plants) screened for the nine most frequent viruses found in Mendoza region (Argentina). The (-) symbol indicates a negative result and (+) symbol indicate positive results. Highlighted in red are positive samples for infrequent viruses in Mendoza region. B) Detailed results of the individual samples that showed positive results in the first screening. [file 12870_2025_6660_MOESM3_ESM.docx]

**Supplementary Table 2**. A) Sanitary status of the 25 Malbec clones analysed, based on composite samples (three plants) screened for the nine most frequent viruses found in Mendoza region (Argentina). The (-) symbol indicates a negative result and (+) symbol indicate positive results. Highlighted in red are positive samples for infrequent viruses in Mendoza region. B) Detailed results of the individual samples that showed positive results in the first screening.

**A**

|  | **Control** | **Screened viruses** | | | | | | | | |
| --- | --- | --- | --- | --- | --- | --- | --- | --- | --- | --- |
| **Clon ID** | **18S** | **GLRaV-1** | **GLRaV-2** | **GLRaV-3** | **GLRaV-4** | **GVA** | **GVB** | **GFkV** | **RSPaV** | **GFLV** |
| 136S | + | - | - | - | - | - | - | - | + | - |
| 42 | + | - | - | - | - | - | - | - | + | - |
| **46** | **+** | **-** | **+** | **-** | **-** | **-** | **-** | **+** | **+** | **-** |
| 501 | + | - | - | - | - | - | - | - | + | - |
| 502 | + | - | - | - | - | - | - | - | + | - |
| 504 | + | - | - | - | - | - | - | - | + | - |
| 505 | + | - | - | - | - | - | - | - | + | - |
| 506 | + | - | - | - | - | - | - | - | + | - |
| 507 | + | - | - | - | - | - | - | - | + | - |
| 508 | + | - | - | - | - | - | - | - | + | - |
| **509** | **+** | **-** | **-** | **-** | **-** | **-** | **-** | **+** | **+** | **-** |
| 510 | + | - | - | - | - | - | - | - | + | - |
| 511 | + | - | - | - | - | - | - | - | + | - |
| 512 | + | - | - | - | - | - | - | - | + | - |
| 513 | + | - | - | - | - | - | - | - | + | - |
| 514 | + | - | - | - | - | - | - | - | + | - |
| 515 | + | - | - | - | - | - | - | - | + | - |
| 595 | + | - | - | - | - | - | - | - | + | - |
| 596 | + | - | - | - | - | - | - | - | + | - |
| **598** | **+** | **-** | **-** | **-** | **-** | **-** | **-** | **+** | **+** | **-** |
| 711 | + | - | - | - | - | - | - | - | + | - |
| **712** | **+** | **-** | **-** | **-** | **-** | **-** | **-** | **+** | **+** | **-** |
| 713 | + | - | - | - | - | - | - | - | + | - |
| 714 | + | - | - | - | - | - | - | - | + | - |
| 715 | + | - | - | - | - | - | - | - | + | - |

**B**

|  | **Control** | **Screened viruses** | | | | | | | | |
| --- | --- | --- | --- | --- | --- | --- | --- | --- | --- | --- |
| **Clon ID** | **18S** | **GLRaV-1** | **GLRaV-2** | **GLRaV-3** | **GLRaV-4** | **GVA** | **GVB** | **GFkV** | **RSPaV** | **GFLV** |
| **46-1** | **+** | **-** | **+** | **-** | **-** | **-** | **-** | **+** | **+** | **-** |
| **46-2** | **+** | **-** | **-** | **-** | **-** | **-** | **-** | **+** | **+** | **-** |
| 46-3 | + | - | - | - | - | - | - | - | - | - |
| **509-1** | **+** | **-** | **-** | **-** | **-** | **-** | **-** | **+** | **+** | **-** |
| 509-2 | + | - | - | - | - | - | - | - | + | - |
| 509-3 | + | - | - | - | - | - | - | - | + | - |
| 598-1 | **+** | **-** | **-** | **-** | **-** | **-** | **-** | **-** | **+** | **-** |
| **598-2** | **+** | **-** | **-** | **-** | **-** | **-** | **-** | **+** | **+** | **-** |
| 598-3 | + | - | - | - | - | - | - | - | + | - |
| **712-1** | **+** | **-** | **-** | **-** | **-** | **-** | **-** | **-** | **+** | **-** |
| **712-2** | **+** | **-** | **-** | **-** | **-** | **-** | **-** | **+** | **+** | **-** |
| 712-3 | + | - | - | - | - | - | - | - | - | - |
