# Supplementary material for: Natural genetic variation for fruit set rate within Malbec grapevine (Vitis vinifera L.) clones
Source: BMC Plant Biol. 2025 May 8;25:606. doi: 10.1186/s12870-025-06660-1 (PMC12060385; doi:10.1186/s12870-025-06660-1)

**Figure S1.** Correlations between manual and automated counting for custom modifications introduced to the semi-automated protocols. Number of flower caps per inflorescence (A), total number of pollen grains per image (B), and viable pollen grains per image (C).


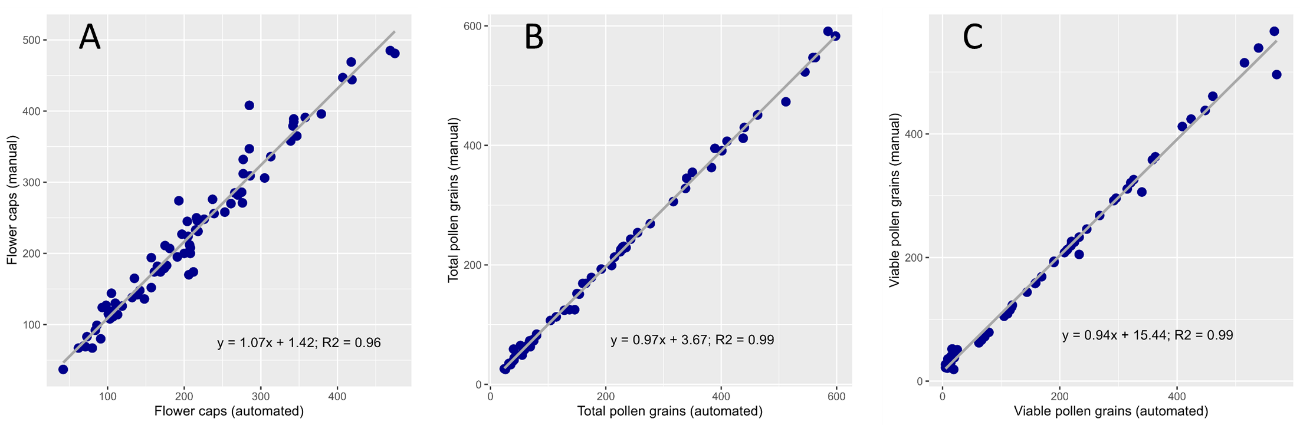

Supplement: Supplementary file 4 — Additional file 4: Figure S1. Correlations between manual and automated counting for custom modifications introduced to the semi-automated protocols. Number of flower caps per inflorescence (A), total number of pollen grains per image (B), and viable pollen grains per image (C). [file 12870_2025_6660_MOESM4_ESM.docx]
