# Supplementary material for: Natural genetic variation for fruit set rate within Malbec grapevine (Vitis vinifera L.) clones
Source: BMC Plant Biol. 2025 May 8;25:606. doi: 10.1186/s12870-025-06660-1 (PMC12060385; doi:10.1186/s12870-025-06660-1)

**Figure S2.** Pearson’s correlation coefficients (r) obtained for the five evaluated traits for 25 clones in two consecutive seasons. Squares size and color vary according to correlation coefficients (see blue-to-red scale in the bottom). White squares indicate non-significant correlation values between variables (*p*-value < 0.05).


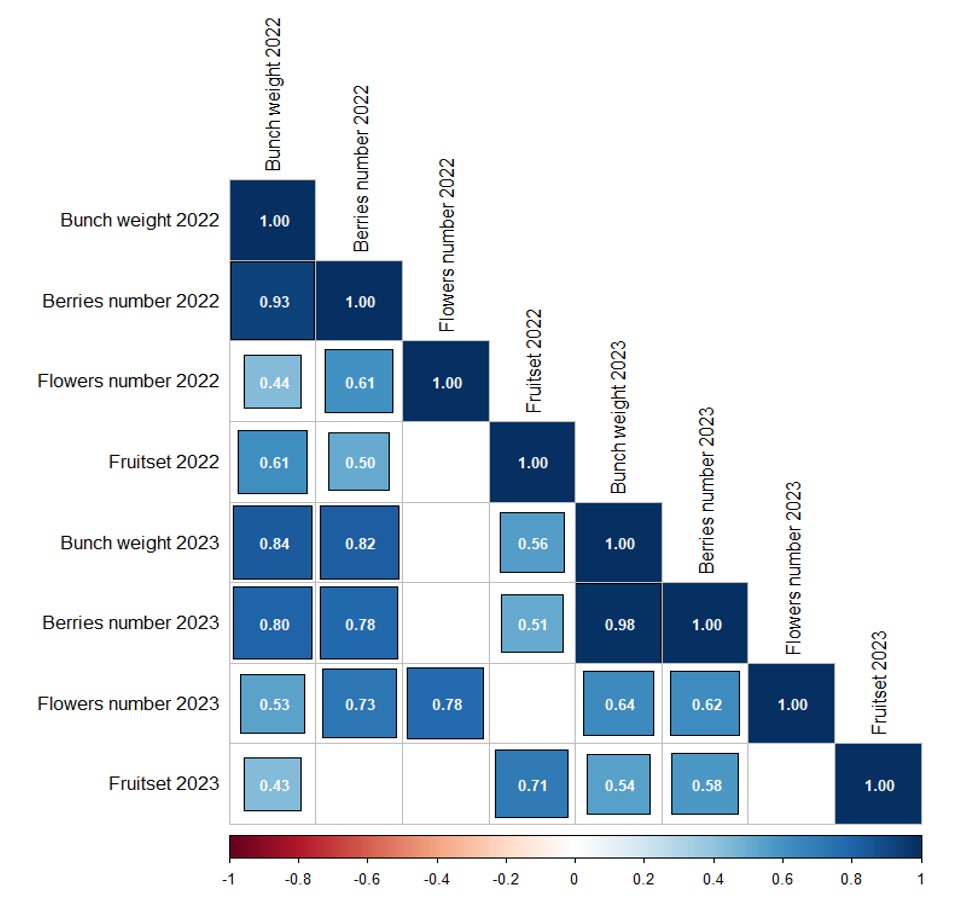

Supplement: Supplementary file 5 — Additional file 5: Figure S2. Pearson’s correlation coefficients (r) obtained for the five evaluated traits for 25 clones in two consecutive seasons. Squares size and color vary according to correlation coefficients (see blue-to-red scale in the bottom). White squares indicate non-significant correlation values between variables (p-value < 0.05). [file 12870_2025_6660_MOESM5_ESM.docx]
