# Supplementary material for: Natural genetic variation for fruit set rate within Malbec grapevine (Vitis vinifera L.) clones
Source: BMC Plant Biol. 2025 May 8;25:606. doi: 10.1186/s12870-025-06660-1 (PMC12060385; doi:10.1186/s12870-025-06660-1)

**Figure S3.** Boxplots showing the phenotypic distribution for bunch weight, based on the clonal groups indicated by HCPC analyses. Groups of clones are colored as indicated in Figure 2. Different lowercase letters indicate significant differences among groups per season (2021/22 and 2022/23), according to a Tukey HSD test (p ≤ 0.05).


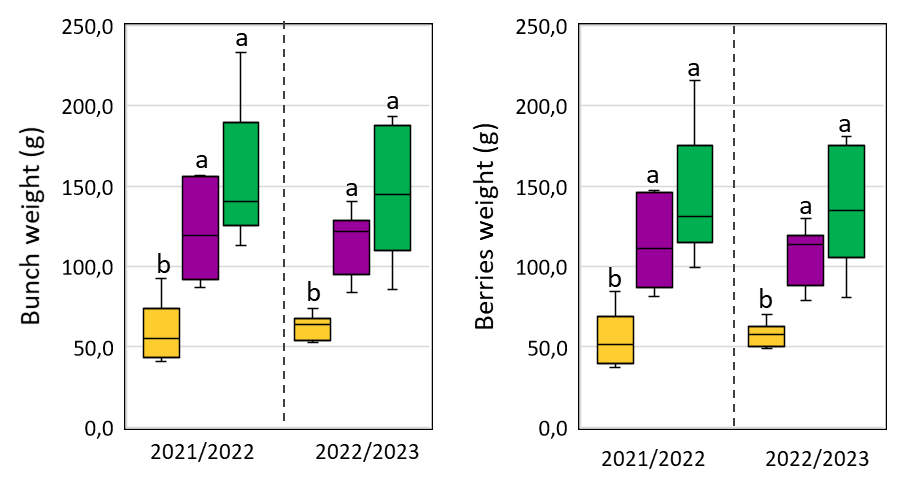

Supplement: Supplementary file 6 — Additional file 6: Figure S3. Boxplots showing the phenotypic distribution for bunch weight, based on the clonal groups indicated by HCPC analyses. Groups of clones are colored as indicated in Fig. 2. Different lowercase letters indicate significant differences among groups per season (2021/22 and 2022/23), according to a Tukey HSD test (p ≤ 0.05). [file 12870_2025_6660_MOESM6_ESM.docx]
