# Supplementary material for: Natural genetic variation for fruit set rate within Malbec grapevine (Vitis vinifera L.) clones
Source: BMC Plant Biol. 2025 May 8;25:606. doi: 10.1186/s12870-025-06660-1 (PMC12060385; doi:10.1186/s12870-025-06660-1)

**Figure S4.** Bar plots showing the pollen viability (%) values obtained for the 25 Malbec clones, during 2023/24. Each bar represents a clone colored according to HPCP results (see Figure 2). Clones in grey showed inconsistent clustering results between seasons. The dashed line indicates the 80% threshold, above which grapevines are considered to have -high to very high- pollen viability. Malbec-510 was the only clone bellow that threshold.


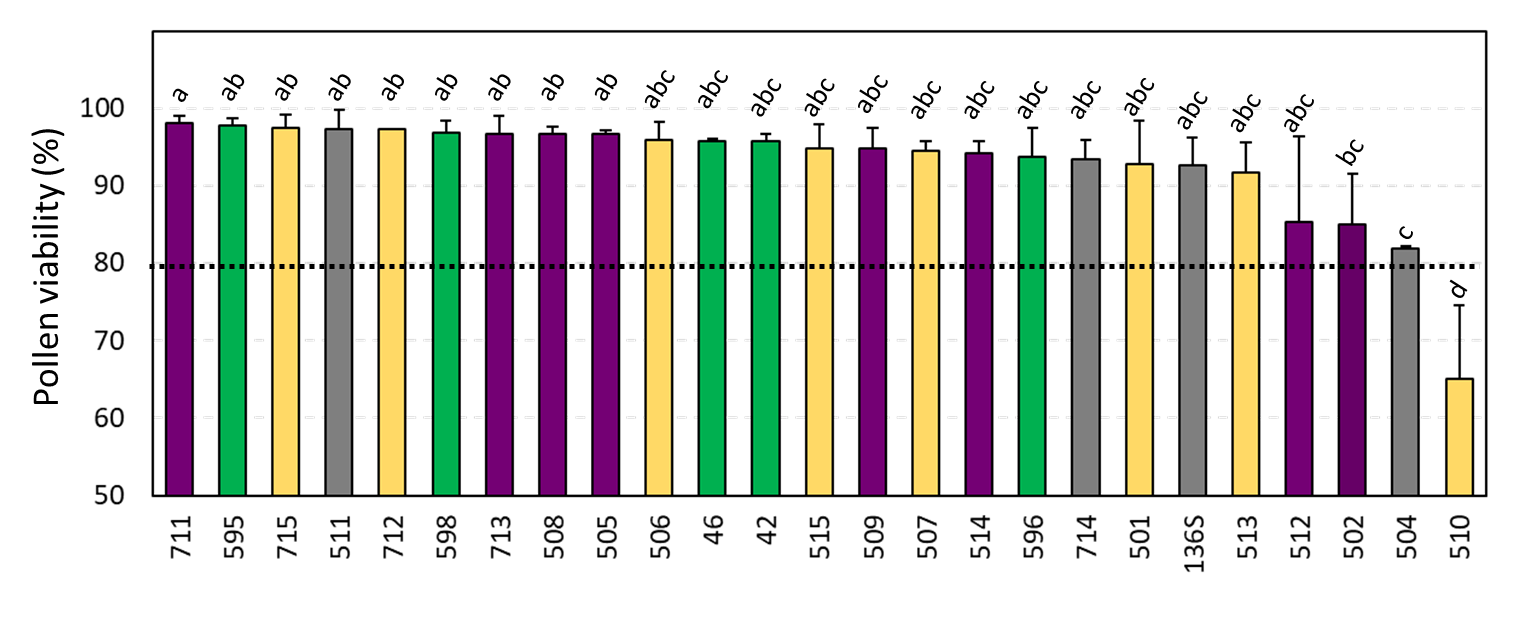

Supplement: Supplementary file 7 — Additional file 7: Figure S4. Bar plots showing the pollen viability (%) values obtained for the 25 Malbec clones, during 2023/24. Each bar represents a clone colored according to HPCP results (see Fig. 2). Clones in grey showed inconsistent clustering results between seasons. The dashed line indicates the 80% threshold, above which grapevines are considered to have -high to very high- pollen viability. Malbec-510 was the only clone below that threshold. [file 12870_2025_6660_MOESM7_ESM.docx]
